# The Role of the TGF-β Coreceptor Endoglin in Cancer

**DOI:** 10.1100/tsw.2010.230

**Published:** 2010-12-14

**Authors:** Eduardo Pérez-Gómez, Gaelle del Castillo, Juan Francisco Santibáñez, Jose Miguel Lêpez-Novoa, Carmelo Bernabéu, Miguel Quintanilla

**Affiliations:** ^1^ Instituto de Investigaciones Biomédicas Alberto Sols, Consejo Superior de Investigaciones Científicas (CSIC)-Universidad Autênoma de Madrid, Madrid, Spain; ^2^ Institute for Medical Research, University of Belgrado, Belgrado, Serbia; ^3^ Instituto Reina Sofía de Investigaciên Nefrolêgica, Departamento de Fisiologia y Farmacología, Universidad de Salamanca, Salamanca, Spain; ^4^ Centro de Investigaciones Biológicas, CSIC, and CIBER de Enfermedades Raras (CIBERER), Madrid, Spain

**Keywords:** endoglin (CD105), TGF-β, angiogenesis, cancer, migration, invasion, malignant progression

## Abstract

Endoglin (CD105) is an auxiliary membrane receptor of transforming growth factor beta (TGF-β) that interacts with type I and type II TGF-β receptors and modulates TGF-β signaling. Endoglin is overexpressed in the tumor-associated vascular endothelium, where it modulates angiogenesis. This feature makes endoglin a promising target for antiangiogenic cancer therapy. In addition, recent studies on human and experimental models of carcinogenesis point to an important tumor cell–autonomous role of endoglin by regulating proliferation, migration, invasion, and metastasis. These studies suggest that endoglin behaves as a suppressor of malignancy in experimental and human epithelial carcinogenesis, although it can also promote metastasis in other types of cancer. In this review, we evaluate the implication of endoglin in tumor development underlying studies developed in our laboratories in recent years.

## INTRODUCTION

The human transforming growth factor beta (TGF-β) family comprises more than 40 structurally related factors, including the classical isoforms TGF-β1, -β2, and -β3; activins; and bone morphogenetic proteins (BMPs); among others. These growth factors play crucial roles in embryonic development, adult tissue homeostasis, and the pathogenesis of a number of diseases[1,2]. TGF-β signals through heteromeric cell-surface type I and II Ser/Thr kinase receptors (TβRI and TβRII) that activate the canonical Smad pathway, as well as Smad-independent signaling pathways, to regulate fundamental cellular processes, such as proliferation, differentiation, migration, programmed cell death, adhesion, cytoskeletal organization,

*Current address: Departamento de Bioquímica y Biología Molecular I, Facultad de Biología, Universidad Complutense de Madrid, Madrid, Spain
**Corresponding author.
©2010 with author.
Published by TheScientificWorld; www.thescientificworld.com extracellular matrix remodeling, and phenotypic plasticity[3,4,5]. Cancer is a multistep process that involves the evolution of a clonal cell population that has escaped from the control of regulatory circuits that govern normal tissue homeostasis. Cancer cells accumulate gain-of-function mutations in oncogenes, which make them independent of growth factor–stimulated proliferation and recessive loss-of-function mutations in tumor suppressors that allow evasion of growth inhibitory signals[6]. TGF-β is a potent growth inhibitor of normal epithelial cells and accumulated evidence indicates that it plays a tumor-suppressive role in epithelial cancer[7]. Thus, tumor cells evade TGF-β growth inhibition either by inactivating components of the TGF-β signaling machinery, including receptors and Smad proteins, or by selectively preventing the TGF-β cytostatic responses[8]. Paradoxically, TGF-β is overexpressed in many human cancers and becomes a promoter of malignancy during tumor progression[9,10,11]. TGF-β drives tumorigenesis by a variety of mechanisms acting on tumor and stromal cells. TGF-β stimulates tumor growth by inducing the production of autocrine mitogenic factors. It also promotes tumor cell migration and invasion by inducing an epithelial-mesenchymal transition (EMT). EMT is a profound phenotypic conversion by which epithelial cells lose their polarity and cohesiveness, acquiring motile and invasive properties. EMT plays a crucial role in morphogenetic processes during organogenesis, but also contributes to pathological situations, such as fibrosis and cancer[12,13]. In the stroma, TGF-β is involved in the generation of activated myofibroblasts from mesenchymal precursors, the stimulation of angiogenesis, the suppression of the immune surveillance, and the promotion of metastasis.

Endoglin is a TGF-β coreceptor that modulates TGF-β–dependent cellular responses. So far, most research studies on endoglin have focused on its role in angiogenesis and vascular remodeling. However, recent data have emerged suggesting that changes of endoglin expression in tumor cells contribute to the deregulation of TGF-β–dependent and –independent signaling pathways and malignant progression.

## ENDOGLIN AND TGF-β SIGNALING

### Endoglin Structure: Isoforms

Endoglin is a type I membrane glycoprotein with a large extracellular domain containing several O- and N-glycans, followed by a hydrophobic membrane-spanning segment and a short cytoplasmic tail. It is expressed at the cell surface as a disulfide-linked homodimer[14,15,16,17]. The extracellular region contains an orphan domain at the N-terminus as well as a zona pellucida (ZP) consensus domain in the juxtamembrane region (Fig. 1a), which is potentially involved in receptor oligomerization[18,19,20]. Within the ZP domain, human endoglin contains an Arg-Gly-Asp (RGD) motif, which is a recognition sequence for integrins[14]. In contrast to the functional TβRI and TβRII receptors, the cytoplasmic domain of endoglin lacks any obvious enzymatic motif, but contains Ser and Thr residues that can be phosphorylated by the TGF-β receptor kinases (see below and Fig. 1c). In addition, the endoglin cytoplasmic tail holds a PDZ-binding motif (Ser-Ser-Met-Ala) at the C-terminus that seems to modulate phosphorylation of the neighboring Ser and Thr residues[21].

At least two distinct alternatively spliced isoforms, long (L)-endoglin and short (S)-endoglin, are expressed in human and mouse tissues[17,22] (see Fig. 1b). L- and S-endoglin only differ from each other in the composition of their cytoplasmic domain. The human L isoform has a cytoplasmic domain of 47 residues, whereas the cytoplasmic tail of the S isoform contains only 14 residues (Fig. 1c). Both endoglin isoforms share the first seven juxtamembrane residues of their cytoplasmic tails, but S-endoglin lacks the PDZ-binding motif[17,22]. Although significant expression of S-endoglin transcripts has been found in some tissues, such as lung and liver[22], L-endoglin seems to be the predominant isoform expressed in most of the tissues, particularly in the endothelium, and is the focus of most studies on endoglin biology. Therefore, when the general term of endoglin is used in this review, it refers to L-endoglin.

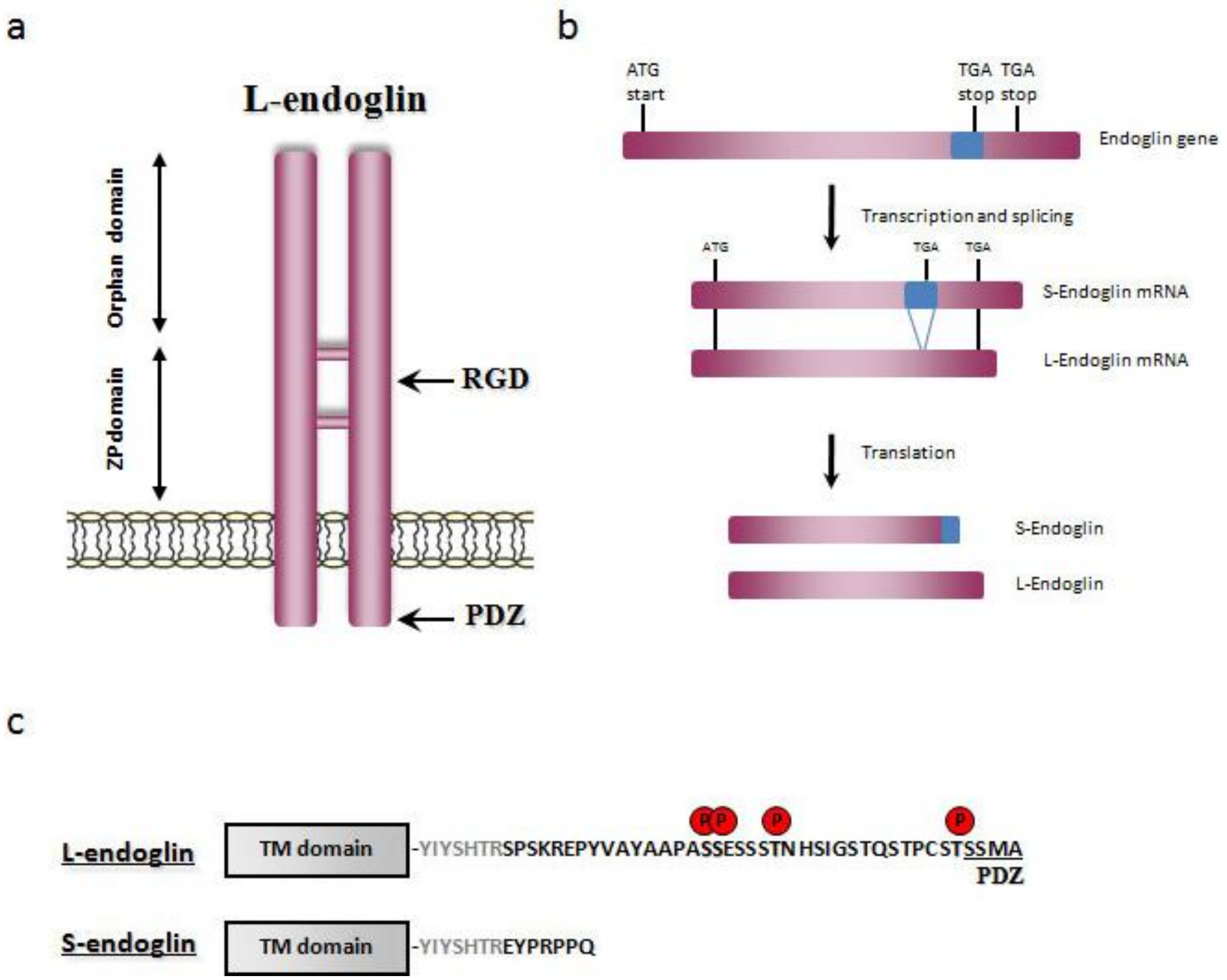

**FIGURE 1**. Endoglin structure and isoforms. (a) Schematic representation of endoglin structure. The location of the integrin-binding RGD motif and PDZ-binding motif are indicated. (b) Diagram representing the alternate splicing mechanism originating L- and S-endoglin. The intron located between exons 14 and 15 (in blue), which contains a TAG stop codon, is transcribed into the S-endoglin mRNA, but is spliced out to yield the L-endoglin transcript. (c) Comparison of human L- and S-endoglin cytoplasmic domains. Identical amino acids are in grey. L-endoglin is phosphorylated at Ser/Thr residues by TβRII, ALK1, and ALK5. TM, transmembrane.

## Endoglin in the TGF-β Receptor Complex

TGF-β growth factors exert their biological effects by binding to a heteromeric complex containing the transmembrane Ser/Thr kinase TβRI and TβRII signaling receptors, although it is TβRI that determines the signaling specificity within the receptor complex[23,24]. The human genome encodes seven TβRI (activin-like kinases or ALKs 1–7) and five TβRII receptors. After binding of the ligand to TβRII, it activates and transphosphorylates TβRI, which subsequently transduces the signal by phosphorylating the receptor-regulated Smads (R-Smads). Activated R-Smads associate with a common homolog named Co-Smad (Smad4 in mammals) and these complexes, then, translocate into the nucleus where they regulate the transcriptional activity of target genes (Fig. 2). TGF-β has been found to activate other signaling mediators in a Smad-independent manner, including several branches of mitogen-activated protein kinase (MAPK) pathways, Rho GTPase signaling pathways, and the phosphatydilinositol-3-kinase (PI3K)/AKT pathway[3,5].

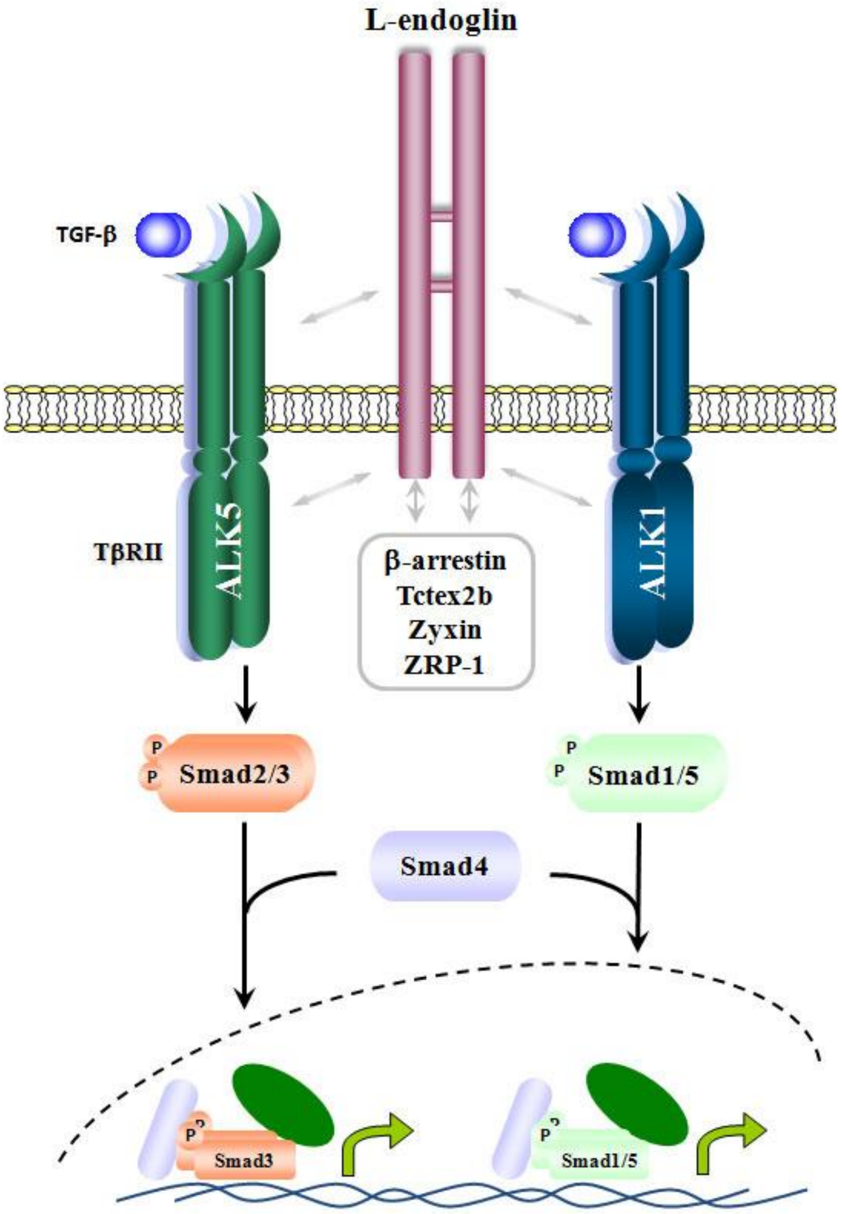

**FIGURE 2**. Hypothetical model for endoglin regulation of TGF-β–dependent and –independent cell responses. Endoglin modulates TGF-β signaling by interacting with TβRI (ALK5 and ALK1) and TβRII through its extracellular and cytoplasmic domains. The endoglin cytoplasmic domain interacts with β-arrestin, Tctex2b, zyxin, and ZRP-1 proteins. Through these interactions, endoglin could mediate F-actin rearrangements, cell adhesion, and migration, as well as protein transport via endocytic vesicles.

The TGF-β receptor complex also contains two auxiliary receptors (or type III receptors), endoglin and betaglycan, that bind different members of the TGF-β family[1,25]. Endoglin and betaglycan share a high degree of amino acid sequence homology in the transmembrane and cytoplasmic domains[26]. At variance with endoglin, no alternatively spliced isoforms have so far been described for betaglycan. Betaglycan has been shown to promote high-affinity binding of TGF-$\beta_2$ to TβRII, since TGF-$\beta_2$ binds

TβRII with low affinity in the absence of betaglycan[27]. Endoglin binds TGF-β1, TGF-β3 (but not TGF-β2), activin-A, BMP-2, and BMP-7 in the presence of the signaling TβRI and TβRII receptors[17,28,29,30]. In contrast, membrane-associated endoglin can bind BMP-9 in the absence of signaling receptors[31]. Activated ALK5 and TβRII interact with the extracellular and cytoplasmic domains of endoglin (Fig. 2). However, unphosphorylated ALK5 only interacts with the endoglin cytoplasmic domain. Upon binding, both ALK5 and TβRII phosphorylate Ser and Thr in the endoglin cytoplasmic domain and, then, ALK5, but not TβRII, dissociates from the receptor complex[32]. On the other hand, Koleva and coworkers have shown that endoglin is a direct substrate of ALK1 in endothelial cells. ALK1 is an endothelial-specific TβRI that preferentially phosphorylates endoglin in Thr residues, although this requires previous Ser phosphorylation by TβRII[21]. Endoglin phosphorylation seems to play a regulatory role for ALK1-dependent endothelial cell growth and adhesion. For a summary of endoglin-binding proteins see Table 1.

**TABLE 1**
**A Summary of Endoglin-Binding Proteins**

| Endoglin Domain | TGF-β Superfamily Ligands | TβRI | TβRII | Other Proteins |
|---|---|---|---|---|
| Extracellular domain | TGF-β1<br>TGF-β3<br>Activin A<br>BMP-2<br>BMP-7<br>BMP-9 | ALK1<br>ALK2<br>ALK3<br>ALK5<br>ALK6 | TβRII<br>ActRII<br>BMPRII | |
| Cytoplasmic domain | | ALK1<br>ALK5 | | Zyxin<br>ZRP-1<br>β-Arrestin2<br>Tctex2β |

These studies indicate that endoglin is part of the ligand-TβRI/TβRII complex, where it likely plays a role in the dynamics of TβRI-TβRII interactions regulating downstream TGF-β signaling. Indeed, a large number of laboratories have reported that endoglin modulates TGF-β–dependent responses in different cell types. Accordingly, endoglin dysfunction may lead to uncontrolled TGF-β signaling activity, often resulting in human disease.

## ENDOGLIN AS A TARGET FOR CANCER IMAGING AND ANTIANGIOGENIC THERAPY

Endoglin is expressed at low levels in resting endothelial cells, but its expression strongly increases in active vascular endothelial cells upon tumor angiogenesis. Endoglin in the blood vessel endothelium is involved in the control of cell proliferation, migration, and capillary tube formation, and plays a proangiogenic role[33,34]. The critical role of endoglin in the vascular system is demonstrated by the identification of *ENG* as the gene mutated in the vascular syndrome Hereditary Hemorrhagic Telangiectasia type I (HHT1)[35]. The finding that endoglin is overexpressed in vascular endothelial cells of tissues undergoing angiogenesis suggested that endoglin detection could be used as a marker to analyze angiogenesis and microvessel density (MVD) in tumors. Increased MVD is associated with bad prognosis in cancer patients, and the use of endoglin antibodies has been found to be the most reliable for MVD measurements when compared to other endothelial protein markers[36,37]. Moreover, its association with actively proliferating endothelial cells, while it is weakly expressed in quiescent endothelium, has focused the interest on endoglin as a potential target for cancer imaging *in vivo*[34]. Studies carried out on animal models[38,39] and patients with renal carcinoma[40] clearly show that antiendoglin monoclonal antibodies specifically target the tumor vasculature. In this respect, endoglin is a more specific marker for new, immature vessels, unlike other endothelial cell-surface markers, such as CD31 and CD34, which are expressed in both mature and immature vessels[41]. The antiendoglin antibodies are useful to detect metastatic tumors that conventional imaging techniques cannot visualize. Also, microbubbles conjugated with an antiendoglin antibody have been successfully utilized to image (by ultrasounds) the effect of gemcitabine on the vasculature in mouse xenografts of human pancreatic tumors[42].

Blocking the tumor blood supply by disrupting or preventing the formation of new blood vessels has been thought a general strategy to treat cancer[43]. The potential of antiendoglin monoclonal antibodies to be used as a therapeutic antiangiogenic strategy in human cancer has received considerable support from preclinical studies. There are excellent reviews addressing this topic[34,37] and, therefore, this matter will not be discussed herein. Nevertheless, it is worth mentioning that a multicenter phase I clinical trial using the antiendoglin antibody TRC105 is ongoing in patients with advanced refractory cancer. The preliminary results support good tolerability and early clinical activity of TRC105[44].

## THE ROLE OF ENDOGLIN IN HUMAN CANCER

Most studies about the involvement of endoglin in cancer have focused on its role as a proangiogenic molecule and its usefulness as a marker of MVD in tumors. However, there is evidence that endoglin modulates cell proliferation, adhesion, and migration of neoplastic cells, suggesting a direct involvement of endoglin in cancer either by modulating the response of tumor cells to TGF-β or by as-yet-not-completely-understood TGF-β–independent mechanisms. In the next sections, we review the current data implicating endoglin in tumor progression, mainly in carcinomas. Endoglin has also been associated with a hereditary cancer disorder known as juvenile polyposis.

### Juvenile Polyposis Syndrome

Juvenile polyposis (JP) is an autosomal-dominant syndrome characterized by multiple hamartomatous polyps occurring throughout the gastrointestinal tract. Patients are usually diagnosed within the first decade, but polyps can appear at any age[45,46]. The term “hamartomatous” was introduced based on the following histological features: dilated cystic glands with retention of mucus and lined by tall columnar epithelium, a markedly expanded lamina propia, and diffuse chronic infiltration of inflammatory cells, to be distinguished from adenomatous polyps[47]. Individuals with JP are at risk of developing gastric, colorectal, and pancreatic malignancies[48,49]. Mutations in either the *MADH4* or *BMPRIA* genes encoding Smad4 and a transmembrane type I receptor for BMPs, respectively, are responsible for the majority of JP cases[45,46]. The overall prevalence of germline mutations is 20% for *MADH4* and 20% for *BMPRIA* in JP probands[50]. *MADH4* mutations are correlated to a high incidence of upper gastrointestinal polyps[51,52]. Some JP patients were observed to develop symptoms of HHT, such as pulmonary arteriovenous malformations (AVMs), which led to the recognition of a combined syndrome, JP-HHT, which is caused by mutations in *MADH4*, but not in the main genes involved in HHT: *ENG* and *ACTIVIN RECEPTOR LIKE KINASE* (*ACVRL1*)[53,54]. JP-HHT patients commonly exhibit AVMs, telangiectases, and epistaxis, as well as gastrointestinal polyps. A recent report has shown that *MADH4* mutations in JP and JP-HHT overlap, indicating that there is no apparent molecular distinction between JP and JP-HHT[55].

Also, germline mutations in *ENG* were found in two out of 14 JP patients who did not contain *MADH4* or *BMPRIA* mutations[56]. Both JP patients had an early onset of the disease (at ages 3 and 5 years) compared to those without *ENG* mutations, and they did not exhibit clinical manifestations or a family history of HHT. The low frequency of *ENG* mutations found in JP patients raised concerns about whether *ENG* is a susceptibility gene for JP. Thus, a subsequent report aimed to examine the prevalence of *ENG* mutations in JP patients who did not have germline mutations in *MADH4* and *BMPRIA* failed to confirm the role of *ENG* as a gene predisposing to JP[57].

## Endoglin Alterations in Human Sporadic Cancer

Only a handful of studies have reported the expression of endoglin in neoplastic cells of primary tumors. In prostate cancer, endoglin was detected in epithelial cells of both prostatic intraepithelial neoplasia and malignant areas, while cells in normal and benign acini were negative[58]. In this study, endoglin staining in prostate cancer cells did not correlate with patient/tumor characteristics, including the levels of the prostatic-specific antigen (PSA) in serum, Gleason score, tumor stage, and periprostatic invasion. In contrast, down-regulation of endoglin expression was found in prostate carcinoma cell lines associated with malignant progression. Suppression of endoglin expression by antisense oligonucleotides enhanced migration and invasion of nontumorigenic prostate cell lines, while overexpression of endoglin had the opposite effect[59]. Moreover, progressive endoglin loss led to progressive increases in the number of circulating prostate carcinoma cells and the formation of metastases[60]. These results suggested a role for endoglin as a modulator of migration, invasiveness, and metastasis in prostate cancer cells, and were in line with the striking observation that only the *ENG* gene among 4,000 genes was found to be down-regulated during detachment of metastatic prostate cancer cells[61].

A report on esophageal squamous cell carcinomas (SCCs) showed that endoglin expression is down-regulated in both primary tumors and cell lines, and that overexpression of endoglin in esophageal SCC cells led to reduced invasiveness and tumorigenicity[62]. The mechanisms for endoglin down-regulation in esophageal tumors include epigenetic silencing by gene methylation and loss of heterozygosity (LOH). Previous studies from the same authors identified that the 9q33-34 region, where the *ENG* gene was mapped[63], is frequently lost in esophageal cancer[64,65], pointing to the existence of a tumor-suppressor gene therein.

Recently, strong evidence pointing to endoglin as a tumor suppressor has been obtained in breast cancer[66]. This report shows that lack of endoglin expression in primary tumors correlates with *ENG* gene methylation and poor clinical outcome. Furthermore, loss of endoglin expression seems to cooperate with the activated ErbB2 oncogene to promote migration and invasion of nontumorigenic breast epithelial cells, while endoglin overexpression reduces the metastatic ability of the aggressive MDA-MB-231 carcinoma cell line. These observations indicate that endoglin acts as a suppressor of invasion and metastasis in breast cancer, and argue against the report of Oxmann and colleagues[67] pointing to a proinvasive rather than suppressor role for endoglin in breast cancer. This latter report shows a direct correlation between enhanced endoglin expression and the metastatic abilities of MDA-MB-231 derived cell lines; however, no study on primary tumors is presented.

Endoglin was also found to be expressed by tumor cells in Ewing sarcoma and melanoma[68]. Endoglin expression in these two types of cancer correlated with tumor cell plasticity; i.e., the ability of tumors to mimic an endothelial-like phenotype and participate in the formation of vascular-like structures (vasculogenic mimicry). Endoglin expression in Ewing sarcoma cells significantly correlated with decreased survival of patients[68], indicating a proinvasive and prometastatic role of endoglin in Ewing tumors. A summary of endoglin alterations found in sporadic human cancer and their correlation with tumor progression is presented in Table 2.

Endoglin expression has been detected by indirect immunofluorescence in hematopoietic malignancies; i.e., 47% of acute myelogenous leukemias (AML), 82% of B-cell acute lymphoblastic leukemias (B-ALL), and 7% of T-cell ALL (T-ALL). Apparently, endoglin is always expressed by the most immature subtypes of acute leukemias and absent in the more differentiated ones[36].

**TABLE 2**
**A Summary of Endoglin Alterations Found in Sporadic Human Cancer and Their Correlation with Tumor Progression**

| Tumor Type | Endoglin Expression | Biological Source | Correlation with/ Comments | Refs. |
|---|---|---|---|---|
| CRC | High levels of Sol-Eng | Serum | Metastasis | [84] |
| Breast carcinoma | High levels of Sol-Eng | Serum/plasma | Metastasis/reduced clinical benefit rate to hormone therapy | [83,84,85] |
| | Down-regulated | Primary tumors | Poor clinical outcome | [66] |
| | Up-regulated | MDA-MB-231–derived cell lines | Metastasis | [67] |
| Prostate carcinoma | Positive in PIN and cancer cells | Primary tumors | No correlation with clinical parameters | [58] |
| | High levels of Sol-Eng | Plasma | Metastasis/PSA recurrence after radical prostatectomy | [87,88] |
| | | Serum/urine | Tumor progression/higher tumor volume | [86] |
| | Down-regulated | Cell lines | Metastasis | [59,60] |
| Esophageal SCC | Down-regulated | Primary tumors and cell lines | Tumor progression | [62] |
| Ewing sarcoma | Up-regulated | Primary tumors and cell lines | Vasculogenic mimicry/decreased survival | [68] |
| Melanoma | Up-regulated | Cell lines | Vasculogenic mimicry | [68] |
| Leukemia | High levels of Sol-Eng | Serum | AML, CML | [89] |

AML, acute myeloid leukemia; CML, chronic myeloid leukemia; CRC, colorectal cancer; Sol-Eng, soluble endoglin; PIN, prostatic intraepithelial neoplasia.

On the other hand, a study in a panel of human tumor cell lines showed that endoglin expression is low in carcinoma cells, while it is high in sarcoma cells. Interestingly, in some carcinoma, melanoma, and osteosarcoma cell lines, the subcellular location of endoglin was the cytoplasm rather than the plasma membrane[69]. The functional significance of this observation remains to be investigated.

## Endoglin Modulates Cell Migration and Invasiveness

It is clear from the above-mentioned studies that endoglin modulates adhesion and migration of cancer cells. This regulatory function of endoglin was also described in different types of nontumorigenic cells, including myoblasts, fibroblasts, and endothelial cells[70,71,72,73,74,75]. Regulation of cell migration by endoglin seems to involve TGF-β–dependent as well as TGF-β–independent mechanisms. Thus, it has been reported that endoglin suppresses invasion and metastasis of prostate carcinoma cells by activating the TβRI ALK2, which in turn activates Smad1[60,76]. Moreover, in prostate carcinoma cells expressing low levels of endoglin, the soy isoflavone genistein inhibited migration and invasiveness by activating ALK2/Smad1, which suggests a high therapeutic benefit for patients with prostate carcinomas expressing low levels of endoglin by treating them with genistein[77]. A recent study suggests, however, that ALK2-mediated phosphorylation of the endoglin cytoplasmic tail also contributes to prostate cancer cell migration through a Smad-independent mechanism[78]. Interestingly, endoglin regulates the expression of integrin β3 and osteopontin, which are required for vasculogenic mimicry in Ewing sarcoma and melanoma cells. Endoglin modulation of tumor cell plasticity in these tumor cells appears to require the concerted action of several signaling pathways, such as BMP, focal adhesion kinase (FAK), and PI3K[68].

Other studies aimed to identify intracellular proteins interacting with the endoglin cytoplasmic domain have reported zyxin and zyxin-related protein 1 (see Table 1), two components of the cytoskeleton located at focal adhesions, as the first cytosolic proteins that bind endoglin[71,72]. Through these protein-protein interactions that could be regulated by phosphorylation[21], endoglin may modulate the organization of the actin cytoskeleton and the adhesive properties of the cells by a TGF-β–independent mechanism. The cytoplasmic domain of endoglin (as well as that of TβRII) also binds Tctex2b, a novel t-complex testis-expressed protein 1/2 (Tctex1/2)–like protein, which is a member of the dynein light chain protein family involved in the transport of cargo, in a retrograde fashion, along the microtubules towards the nucleus[79]. Also, an interesting mechanism involving endoglin modulation of epithelial cell adhesive activity has been found during bacterial colonization of mucosal surfaces. Carcinoembryonic antigen (CEA)–binding bacteria trigger *de novo* expression of endoglin, which leads to changes in the composition of focal adhesions and the activation of β1 integrins, suppressing exfoliation of mucosal cells, which protects the mucosa from bacterial infection[80]. Therefore, endoglin regulates the expression and activity/organization of cell adhesion and cytoskeletal components by as-yet-unknown mechanisms.

## Soluble Endoglin in Cancer

The extracellular domain of membrane-bound endoglin can be proteolytically cleaved, releasing a circulating form of endoglin named soluble endoglin (Sol-Eng). Increased levels of Sol-Eng are linked to the pathogenesis of severe vascular diseases, such as systemic sclerosis and pre-eclampsia[81,82]. Also, a number of laboratories have found elevated levels of Sol-Eng in body fluids from cancer patients with respect to healthy donors (Table 2 and Fig. 3). Increased Sol-Eng levels correlate with metastasis in breast cancer and colorectal cancer[83,84]. Sol-Eng levels were decreased in patients receiving chemotherapy, which reduces the utility of Sol-Eng as a prognostic marker to the long-term follow-up of cancer survivors who are not treated with chemotherapy[84]. Elevated levels of plasma Sol-Eng also predict decreased response and survival to hormone therapy of women with metastatic breast cancer[85]. In prostate cancer, high levels of serum Sol-Eng correlate with advanced stages of tumor progression and urinary Sol-Eng seems to be a useful marker for diagnosis[86]. In addition, recent reports have shown that determination of plasma Sol-Eng in prostate cancer has a predictive value for metastasis to the pelvic lymph nodes[87], as well as for increased risk of PSA recurrence in patients treated with radical prostatectomy and bilateral pelvic lymphadenectomy[88]. High levels of Sol-Eng are also present in myeloid malignancies that are characterized by a high cellular proliferation rate, such as acute myeloid leukemia and chronic myeloproliferative disorders[89]. Nonetheless, other reports on gastric, esophageal, and ovarian tumors failed to find Sol-Eng as a valuable marker in the assessment of cancer spread[90,91,92,93,94].

Sol-Eng contains the binding site for different members of the TGF-β superfamily and may act as a scavenger of circulating ligands, preventing their binding to the functional receptors. Thus, Sol-Eng impairs TGF-β signaling, interfering with vascular function and inhibiting angiogenesis[82,95]. An intriguing question concerns the source of Sol-Eng detected in cancer patients. Since endoglin levels are higher in tumor vessels than in tumor cells, it is thought that Sol-Eng derives from endoglin shedding in vascular endothelial cells and that it may represent a surrogate marker of angiogenic activity[96]. However, our studies in an experimental model of carcinogenesis (see below) suggest that endoglin shedding in tumor cells can also contribute to increased levels of Sol-Eng (Fig. 3).

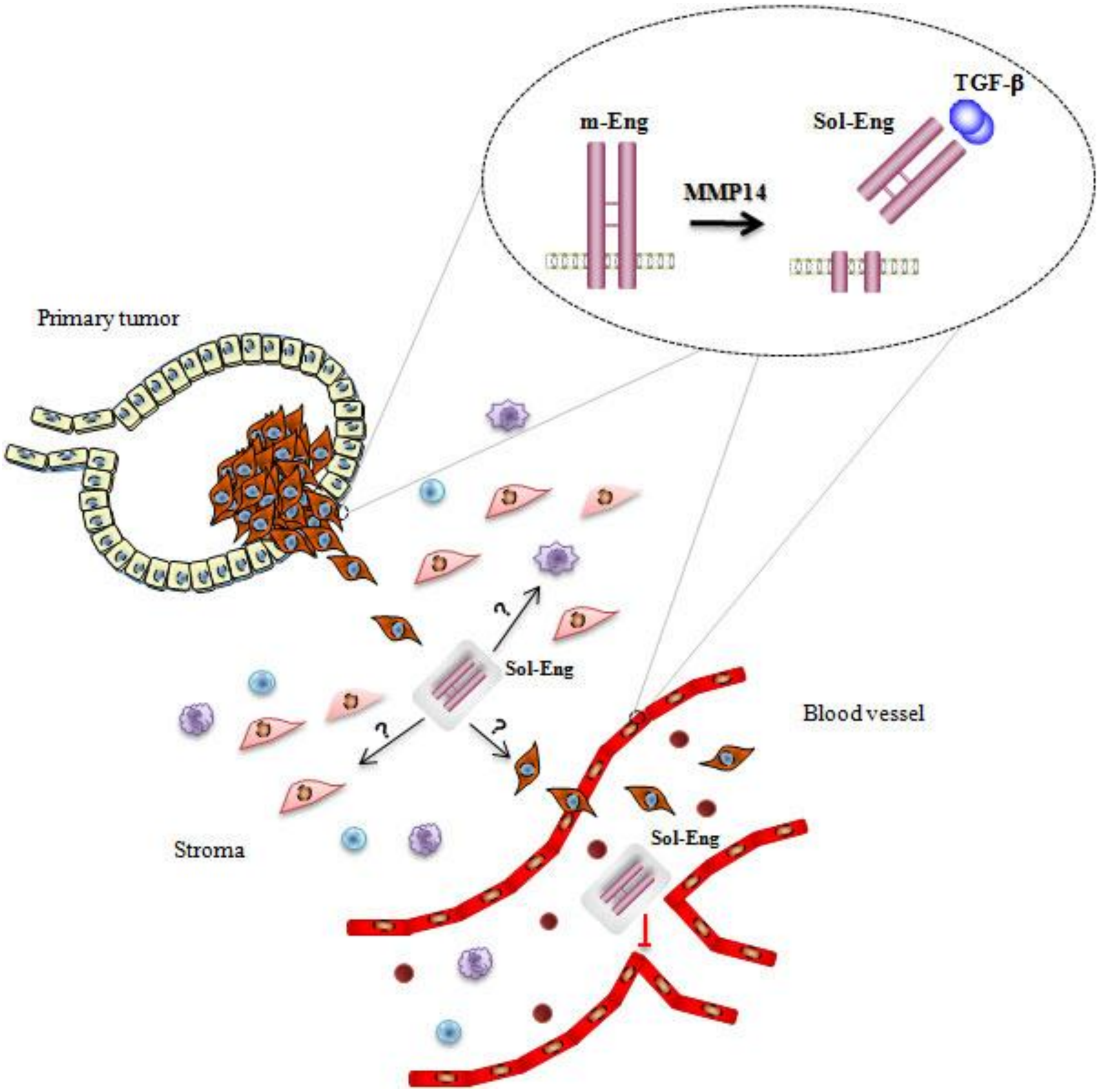

**FIGURE 3**. Generation of Sol-Eng in tumors and its possible actions. The proteolytic cleavage of membrane endoglin (m-Eng) to release Sol-Eng can occur in the primary tumor as well as in the stroma and blood vessels. It has been shown that Sol-Eng inhibits angiogenesis, but its effects on the behavior of tumor cells, cancer-associated fibroblasts (pink), and inflammatory cells (blue and purple) have not yet been tested.

On the other hand, the correlation between elevated Sol-Eng levels and poor clinical outcome of cancer patients is paradoxical, as Sol-Eng seems to be an antiangiogenic agent. In this respect, recent epidemiological studies report that formerly pre-eclamptic women have a reduced incidence of breast cancer. The mechanism underlying this observation is unknown, but as pre-eclampsia is linked to an imbalance between proangiogenic (such as VEGF) and antiangiogenic factors, such as Sol-Eng and soluble fms-like tyrosine kinase-1 (sFlt-1), persistent mild elevations of antiangiogenic Sol-Eng and sFlt-1 may contribute to the inhibition of tumor development later in life[97].

Recently, it has been reported that the membrane-type matrix metalloproteinase-14 (MMP-14, also called MT1-MMP) is involved in Sol-Eng production[95]. This study shows that efficient endoglin shedding requires a direct interaction of endoglin and MMP-14, suggesting that the origin of Sol-Eng in cancer resides in those sites where endoglin and MMP-14 are colocalized (Fig. 3).

## ENDOGLIN IN EXPERIMENTAL CARCINOGENESIS

### The Two-Stage Mouse Skin Carcinogenesis Model

Two-stage mouse skin chemical carcinogenesis has become one of the best established *in vivo* models to study the sequential development of tumors[98,99,100]. Concepts such as tumor initiation, promotion, and progression emerged from this model, which has been classically utilized in environmental cancer studies to test chemicals that may cause, prevent, or cure cancer[101]. Nowadays, the mouse skin chemical carcinogenesis system is also used to evaluate the impact of genetic background and genetic manipulation on tumorigenesis. Tumor induction in two-stage carcinogenesis involves a single subcarcinogenic dose of a carcinogen initiator, such as 7,12-dimethylbenz(a)anthracene (DMBA). This event alone does not give rise to tumors unless followed by repeated application of a tumor promoter, such as 12-O-tetradecanoylphorbol-13-acetate (TPA). This protocol gives rise to the appearance of multiple benign papillomas representing clonal outgrowths of epidermal keratinocytes with initiating mutations in the *Hras1* gene[102]. Papillomas can progress to malignant SCCs; however, there is evidence that papillomas are heterogeneous in their potential for malignant conversion. Thus, two entities named as low- and high-risk benign papillomas with differing rates of premalignant progression have been defined[103]. SCCs are highly vascularized, inward invasive lesions that can be classified into well-differentiated (grade I), moderately differentiated (grade II), and poorly differentiated (grade III/IV) according to the degree of squamous differentiation[104]. This latter group includes the highly aggressive spindle cell carcinoma (SpCC) type of tumor formed by elongated or spindle-shaped cells that have elicited an irreversible EMT[105] (Fig. 4). Progression from papillomas to SCCs is associated with trisomies of chromosomes 6 and 7 (the *Hras1* gene is located on chromosome 7), as well as inactivating mutations in *Trp53* (encoding the tumor suppressor p53) and increasing aneuploidy and dysplasia[98,105]. The SCC-SpCC transition correlates with increased ratio of oncogenic vs. normal *Hras1* expression, and loss or misregulation of the *Ink4* locus encoding the cell cycle inhibitors p16Ink4a, p15Ink4b, and p19Arf[105]. Interestingly, p15Ink4b mediates the growth inhibitory response induced by TGF-β1 in epithelial cells[8], and SpCC cells do not respond to TGF-β–induced growth inhibition.

TGF-β is an important regulator of epidermal homeostasis. TGF-β was documented to inhibit proliferation, but promote differentiation of keratinocytes, suggesting that it primarily acts as a tumor suppressor in skin carcinogenesis[106]. Indeed, a biphasic role for TGF-β1 as a suppressor and a promoter of carcinogenesis (see Fig. 4) was demonstrated in transgenic mice overexpressing the growth factor in differentiating epidermal keratinocytes[107]. TGF-β1 is thought to inhibit proliferation and induce apoptosis during initiation and early stages of carcinogenesis. At later stages, elevated levels of TGF-β1 stimulate malignant progression by promoting tumor cell migration, invasion, and metastasis in a cell-autonomous manner, and by influencing the tumor micoenvironment, facilitating angiogenesis and evasion of the immune system[9,10]. TGF-β1 seems to be the physiological agent involved in pushing the SCC-SpCC transition during mouse skin carcinogenesis, likely in cooperation with the *Hras1* oncogene[105].

### Endoglin Studies on Mouse Skin Carcinogenesis

When $Eng^{+/-}$ heterozygous mice ($Eng^{-/-}$ null embryos die at mid-gestation) were subjected to two-stage skin carcinogenesis with DMBA and TPA, these animals developed a lower number of tumors than control $Eng^{+/+}$ mice, but the frequency of papilloma-carcinoma conversion and the incidence of poorly differentiated SCCs was vastly increased[22,108]. Accelerated malignant progression in $Eng^{+/-}$ mice

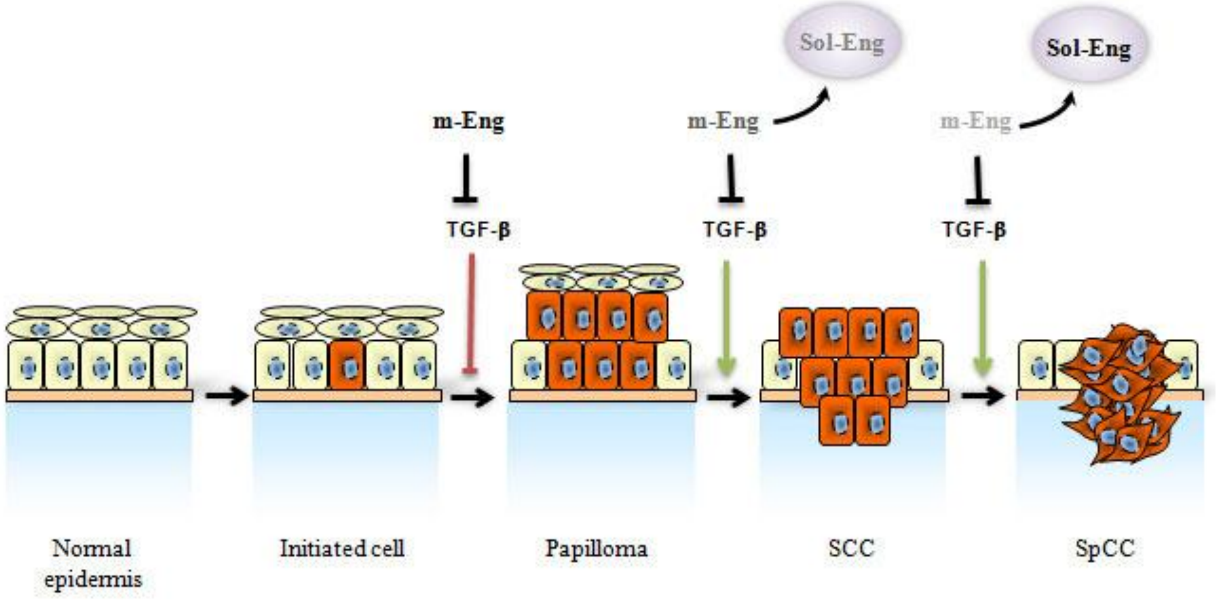

**FIGURE 4**. Schematic representation of the different stages of chemical mouse skin carcinogenesis. The keratinocyte in the epidermis colored in brown represents a cell with *Hras1* mutation initiated by DMBA. Mutated *Hras1* gene expression increases during tumor progression. TGF-β1 behaves as a tumor suppressor at early stages of carcinogenesis, but promotes tumor progression at later stages. Proteolysis of m-Eng to produce Sol-Eng mainly occurs during the transition from SCCs to SpCCs. m-Eng, which inhibits TGF-β signaling in keratinocytes, behaves as a suppressor of malignancy at later stages of carcinogenesis.

occurred in adverse conditions, as these animals exhibit a deficient angiogenic switch[109]. The carcinogenic phenotype of $Eng^{+/-}$ mice was identical to that of mice with targeted expression of TGF-β1 to the epidermis[107], mirroring the dual role of TGF-β1 in carcinogenesis as a tumor suppressor and promoter. These results suggested that endoglin contributes to epidermal homeostasis by attenuating the growth inhibitory response elicited by TGF-β in basal keratinocytes, which is the compartment expressing endoglin in the normal epidermis[108]. This hypothesis was confirmed by demonstrating that endoglin indeed inhibits the TGF-β/ALK5/Smad2/3 signaling pathway in keratinocytes[110]. In addition, the tumor phenotype of $Eng^{+/-}$ mice pointed to a role for endoglin as a suppressor of malignancy at late stages of skin carcinogenesis.

The analysis of endoglin expression in chemically induced mouse skin tumors and cell lines revealed a novel mechanism for inactivation of endoglin in tumor cells by shedding. The truncation of membrane-associated endoglin releasing Sol-Eng into the stroma and blood stream was associated with the SCC-SpCC transition[110] (Fig. 4). Endoglin expression studies also showed that the S-endoglin isoform is neither expressed in normal skin nor tumors, and consequently does not play any role in skin carcinogenesis.

The functional consequences of inactivating membrane endoglin in tumor cells were approached by using short hairpin RNA (shRNA) interfering technology. Down-regulation of endoglin expression in cultured SCC cells induced the constitutive activation of the ALK5/Smad2/3 signaling pathway independently of ligand binding, leading to EMT and a SCC-SpCC conversion *in vivo*[110]. In this respect, it should be mentioned that a role for endoglin in embryonic EMT during cardiac valve formation was also simultaneously reported[111]. These results unequivocally demonstrated that endoglin acts as a suppressor of malignancy during mouse skin carcinogenesis. Likewise, since endoglin knockdown in SCC cells was also linked to reduced cell growth and delayed tumor latencies, we postulated that inactivation of membrane endoglin in tumor cells exerts a strong selective pressure during SCC-SpCC

progression by priming cells that have lost the TGF-β antiproliferative response. This could be the reason why loss of the TGF-β growth inhibitory response cosegregates with the spindle tumor phenotype[112].

Recent results suggest a novel regulatory function for endoglin on mouse skin carcinogenesis. TGF-β1 stimulates *Hras1* gene expression in transformed keratinocytes by a Smad-independent mechanism involving the Ras/ERK signaling pathway. Interestingly, endoglin not only inhibits basal and TGF-β–mediated ERK signaling activity attenuating TGF-β–induced *Hras1* gene expression, but also protects cells from *Hras* oncogene transformation[113]. This finding unveils a new aspect for the suppressor role of endoglin during mouse skin carcinogenesis, a model in which *Hras1* activation drives not only tumor initiation, but also malignant progression[99,105].

## CONCLUSIONS AND FUTURE DIRECTIONS

Endoglin, by virtue of its proangiogenic activity, has become a well-established marker to analyze angiogenesis and MVD in tumors. It also has revealed its potential as a target for cancer imaging and antiangiogenic therapy. New insights have emerged recently on the role of endoglin as a tumor suppressor or promoter during progression to malignancy. However, a greater effort is needed to unravel the clinical significance of endoglin expression in human primary tumors by studying larger cohorts of patients and using a better classification of tumors. Whereas some reports suggest that the basis for the involvement of endoglin in tumor cell migration and invasiveness resides in its ability to modulate TGF-β signaling, in most cases, the precise mechanism for which endoglin regulates these processes is unknown. In particular, studies on the interaction of endoglin with components of the cytoskeleton and the cell adhesion machinery, and on its regulation of TGF-β–independent cell responses, should be continued.

The assigned role of endoglin as a suppressor of malignancy in epithelial cancer (the most common cancer type in humans) raises important questions for HHT patients, who inherit an inactivated *ENG* allele by the germline. The studies on $Eng^{+/-}$ mice predict that HHT1 patients might be protected for tumor formation, but tumors arising in these individuals will be prone to rapid progression to malignant carcinomas. Nonetheless, epidemiological data for cancer incidence in HHT1 patients are lacking at the present time.

Further investigations are also required in order to solve the contradiction of Sol-Eng as a bad prognostic factor in some types of human cancer, while having an antiangiogenic function in pre-eclampsia and other vascular diseases. An explanation for this paradox might be that production of Sol-Eng is a late event during carcinogenesis (see Fig. 4) and, therefore, its antiangiogenic activity may be irrelevant for tumor growth at this stage. Nevertheless, it should be of interest to study the effects of Sol-Eng on tumor cell behavior and on the competence of stromal cells (cancer-associated fibroblasts [CAFs], inflammatory cells) to sustain tumor growth (Fig. 3). On the other hand, there are no studies addressing the role of membrane-associated endoglin expressed in the stromal compartment. In particular, whether endoglin regulates the activity of CAFs to promote tumor growth and progression should be investigated in the next years.

## ACKNOWLEDGMENTS

The work developed in our laboratories is supported by grants from the Spanish Ministry of Science and Innovation (SAF2007-63821 to MQ, SAF2007-63893 to JML-N, and SAF2007-61827 to CB), the *Instituto de Salud Carlos III* (ISCIII-CIBER CB/06/07/0038 to CB and ISCIII-RD06/0016 to JML-N), and *Centro de Investigación Biomédica en Red de Enfermedades Raras* (CIBERER). The CIBER of *Enfermedades Raras* (Rare Diseases) is an initiative of the *Instituto de Salud Carlos III* (ISCIII) of Spain. EP-G is funded by a contract from the Scientific Foundation of the *Asociación Española contra el Cáncer*, and GC is the recipient of a contract from the *Juan de la Cierva* program of the Spanish Ministry of Science and Innovation. We apologize to those colleagues whose work has not been included in this review due to space limitations.

## References

1. Gordon, K.J. and Blobe, G.C. (2008) Role of transforming growth factor-beta superfamily signaling pathways in human disease. *Biochim. Biophys. Acta* **1782**, 197–228.
2. Padua, D. and Massague, J. (2009) Roles of TGF beta in metastasis. *Cell Res*. **19**, 89–102.
3. Derynck, R. and Zhang, Y.E. (2003) Smad-dependent and Smad-independent pathways in TGF-beta family signalling. *Nature* **425**, 577–584.
4. Zavadil, J. and Bottinger, E.P. (2005) TGF-beta and epithelial-to-mesenchymal transitions. *Oncogene* **24**, 5764–5774.
5. Zhang, Y.E. (2009) Non-Smad pathways in TGF-beta signaling. *Cell Res*. **19**, 128–139.
6. Hahn, W.C. and Weinberg, R.A. (2002) Modelling the molecular circuitry of cancer. *Nat. Rev. Cancer* **2**, 331–341.
7. Levy, L. and Hill, C.S. (2006) Alterations in components of the TGF-beta superfamily signaling pathways in human cancer. *Cytokine Growth Factor Rev.* **17**, 41–58.
8. Seoane, J. (2006) Escaping from the TGF-beta anti-proliferative control. *Carcinogenesis* **27**, 2148–2156.
9. Derynck, R., Akhurst, R.J., and Balmain, A. (2001) TGF-beta signaling in tumor suppression and cancer progression. *Nat. Genet*. **29**, 117–129.
10. Massague, J. (2008) TGF beta in cancer. *Cell* **134**, 215–230.
11. Ikushima, H. and Miyazono, K. (2010) TGF beta signalling: a complex web in cancer progression. *Nat. Rev. Cancer* **10**, 415–424.
12. Thiery, J.P., Acloque, H., Huang, R.Y.J., and Nieto, M.A. (2009) Epithelial-mesenchymal transitions in development and disease. *Cell* **139**, 871–890.
13. Lopez-Novoa, J.M. and Nieto, M.A. (2009) Inflammation and EMT: an alliance towards organ fibrosis and cancer progression. *EMBO Mol. Med*. **1**, 303–314.
14. Gougos, A. and Letarte, M. (1990) Primary structure of endoglin, an RGD-containing glycoprotein of human endothelial cells. *J. Biol. Chem*. **265**, 8361–8364.
15. Gougos, A., St Jacques, S., Greaves, A., Oconnell, P.J., D'Apice, A.J.F., Buhring, H.J., Bernabeu, C., Vanmourik, J.A., and Letarte, M. (1992) Identification of distinct epitopes of endoglin, an RGD-containing glycoprotein of endothelial-cells, leukemic-cells, and syncytiotrophoblasts. *Int. Immunol*. **4**, 83–92.
16. Moren, A., Ichijo, H., and Miyazono, K. (1992) Molecular cloning and characterization of the human and porcine transforming growth-factor-beta type-III Receptors. *Biochem. Biophys. Res. Commun.* **189**, 356–362.
17. Bellon, T., Corbi, A., Lastres, P., Cales, C., Cebrian, M., Vera, S., Cheifetz, S., Massague, J., Letarte, M., and Bernabeu, C. (1993) Identification and expression of 2 forms of the human transforming growth factor-beta-binding protein endoglin with distinct cytoplasmic regions. *Eur. J. Immunol*. **23**, 2340–2345.
18. Bork, P. and Sander, C. (1992) A large domain common to sperm receptors (Zp2 and Zp3) and TGF-beta type-III receptor. *FEBS Lett.* **300**, 237–240.
19. Jovine, L., Darie, C.C., Litscher, E.S., and Wassarman, P.M. (2005) Zona pellucida domain proteins. *Annu. Rev. Biochem*. **74**, 83–114.
20. Llorca, O., Trujillo, A., Blanco, F.J., and Bernabeu, C. (2007) Structural model of human endoglin, a transmembrane receptor responsible for hereditary hemorrhagic telangiectasia. *J. Mol. Biol*. **365**, 694–705.
21. Koleva, R.I., Conley, B.A., Romero, D., Riley, K.S., Marto, J.A., Lux, A., and Vary, C.P.H. (2006) Endoglin structure and function -- determinants of endoglin phosphorylation by transforming growth factor-beta receptors. *J. Biol. Chem*. **281**, 25110–25123.
22. Perez-Gomez, E., Eleno, N., Lopez, J.M., Ramirez, J.R., Velasco, B., Letarte, M., Bernabeu, C., and Quintanilla, M. (2005) Characterization of murine S-endoglin isoform and its effects on tumor development. *Oncogene* **24**, 4450–4461.
23. Feng, X.H. and Derynck, R. (2005) Specificity and versatility in TGF-beta signaling through Smads. *Annu. Rev. Cell Dev. Biol*. **21**, 659–693.
24. Massague, J. and Gomis, R.R. (2006) The logic of TGF beta signaling. *FEBS Lett*. **580**, 2811–2820.
25. Massague, J. (1998) TGF-beta signal transduction. *Annu. Rev. Biochem*. **67**, 753–791.
26. Lopez-Casillas, F., Cheifetz, S., Doody, J., Andres, J.L., Lane, W.S., and Massague, J. (1991) Structure and expression of the membrane proteoglycan betaglycan, a component of the TGF-beta receptor system. *Cell* **67**, 785–795.
27. Esparza-Lopez, J., Montiel, J.L., Vilchis-Landeros, M.M., Okadome, T., Miyazono, K., and Lopez-Casillas, F. (2001) Ligand binding and functional properties of betaglycan, a co-receptor of the transforming growth factor-beta superfamily -- specialized binding regions for transforming growth factor-beta and inhibin A. *J. Biol. Chem*. **276**, 14588–14596.

28. Cheifetz, S., Bellon, T., Cales, C., Vera, S., Bernabeu, C., Massague, J., and Letarte, M. (1992) Endoglin is a component of the transforming growth factor-beta receptor system in human endothelial cells. *J. Biol. Chem*. **267**, 19027–19030.
29. Letamendia, A., Lastres, P., Botella, L.M., Raab, U., Langa, C., Velasco, B., Attisano, L., and Bernabeau, C. (1998) Role of endoglin in cellular responses to transforming growth factor beta -- a comparative study with betaglycan. *J. Biol. Chem*. **273**, 33011–33019.
30. Barbara, N.P., Wrana, J.L., and Letarte, M. (1999) Endoglin is an accessory protein that interacts with the signaling receptor complex of multiple members of the transforming growth factor-beta superfamily. *J. Biol. Chem*. **274**, 584–594.
31. Scharpfenecker, M., van Dinther, M., Liu, Z., van Bezooijen, R.L., Zhao, Q.H., Pukac, L., Lowik, C.W.G.M., and ten Dijke, P. (2007) BMP-9 signals via ALK1 and inhibits bFGF-induced endothelial cell proliferation and VEGF-stimulated angiogenesis. *J. Cell Sci*. **120**, 964–972.
32. Guerrero-Esteo, M., Sanchez-Elsner, T., Letamendia, A., and Bernabeu, C. (2002) Extracellular and cytoplasmic domains of endoglin interact with the transforming growth factor-beta receptors I and II. *J. Biol. Chem*. **277**, 29197–29209.
33. Bernabeu, C., Conley, B.A., and Vary, C.P.H. (2007) Novel biochemical pathways of endoglin in vascular cell physiology. *J. Cell. Biochem*. **102**, 1375–1388.
34. Fonsatti, E., Nicolay, H.J.M., Altomonte, M., Covre, A., and Maio, M. (2010) Targeting cancer vasculature via endoglin/CD105: a novel antibody-based diagnostic and therapeutic strategy in solid tumours. *Cardiovasc. Res*. **86**, 12–19.
35. Lebrin, F. and Mummery, C.L. (2008) Endoglin-mediated vascular remodeling: mechanisms underlying hereditary hemorrhagic telangiectasia. *Trends Cardiovas. Med*. **18**, 25–32.
36. Fonsatti, E., Del Vecchio, L., Altomonte, M., Sigalotti, L., Nicotra, M.R., Coral, S., Natali, P.G., and Maio, M. (2001) Endoglin: an accessory component of the TGF-beta-binding receptor-complex with diagnostic, prognostic, and bioimmunotherapeutic potential in human malignancies. *J. Cell. Physiol*. **188**, 1–7.
37. Bernabeu, C., Lopez-Novoa, J.M., and Quintanilla, M. (2009) The emerging role of TGF-beta superfamily coreceptors in cancer. *Biochim. Biophys. Acta* **1792**, 954–973.
38. Fonsatti, E., Jekunen, A.P., Kairemo, K.J.A., Coral, S., Snellman, M., Nicotra, M.R., Natali, P.G., Altomonte, M., and Maio, M. (2000) Endoglin is a suitable target for efficient imaging of solid tumors: In vivo evidence in a canine mammary carcinoma model. *Clin. Cancer Res.* **6**, 2037–2043.
39. Bredow, S., Lewin, M., Hofmann, B., Marecos, E., and Weissleder, R. (2000) Imaging of tumour neovasculature by targeting the TGF-beta binding receptor endoglin. *Eur. J. Cancer* **36**, 675–681.
40. Costello, B., Chenggang, L.G., Duff, S., Butterworth, D., Khan, A., Perkins, M., Owens, S., Al-Mowallad, A.F., O'Dwyer, S.O., and Kumar, S. (2004) Perfusion of Tc-99(m)-labeled CD105 Mab into kidneys from patients with renal carcinoma suggests that CD105 is a promising vascular target. *Int. J. Cancer* **109**, 436–441.
41. Beresford, M.J., Harris, A.L., Ah-See, M., Daley, F., Padhani, A.R., and Makris, A. (2006) The relationship of the neo-angiogenic marker, endoglin, with response to neoadjuvant chemotherapy in breast cancer. *Br. J. Cancer* **95**, 1683–1688.
42. Korpanty, G., Grayburn, P.A., Shohet, R.V., and Brekken, R.A. (2005) Targeting vascular endothelium with avidin microbubbles. *Ultrasound Med. Biol*. **31**, 1279–1283.
43. Folkman, J. (1996) New perspectives in clinical oncology from angiogenesis research. *Eur. J. Cancer* **32A**, 2534–2539.
44. Seon, B.K., Haba, A., Matsuno, F., Takahashi, N., Tsujie, M., She, X., Harada, N., Uneda, S., Tsujie, T., Toi, H., Tsai, H., Haruta, Y. (2010) Endoglin-targeted cancer therapy. *Curr. Drug Deliv.* [Epub ahead of print]
45. Zbuk, K.M. and Eng, C. (2007) Hamartomatous polyposis syndromes. *Nat. Clin. Pract. Gastr. Hepatol.* **4**, 492–502.
46. Calva, D. and Howe, J.R. (2008) Hamartomatous polyposis syndromes. *Surg. Clin. North Am.* **88**, 779–817.
47. Horrilleno, E.G., Eckert, C., and Ackerman, L.V. (1957) Polyps of the rectum and colon in children. *Cancer* **10**, 1210–1220.
48. Howe, J.R., Mitros, F.A., Summers, R.W. (1998) The risk of gastrointestinal carcinoma in familial juvenile polyposis. *Ann. Surg. Oncol*. **5**, 751–756.
49. Brosens, L.A.A., van Hattem, A., Hylind, L.M., Iacobuzio-Donahue, C., Romans, K.E., Axilbund, J., Cruz-Correa, M., Tersmette, A.C., Offerhaus, G.J.A., and Giardiello, F.M. (2007) Risk of colorectal cancer in juvenile polyposis. *Gut* **56**, 965–967.
50. Howe, J.R., Sayed, M.G., Ahmed, A.F., Ringold, J., Larsen-Haidle, J., Merg, A., Mitros, F.A., Vaccaro, C.A., Petersen, G.M., Giardiello, F.M., Tinley, S.T., Aaltonen, L.A., and Lynch, H.T. (2004) The prevalence of MADH4 and BMPR1A mutations in juvenile polyposis and absence of BMPR2, BMPR1B, and ACVR1 mutations. *J. Med. Genet*. **41**, 484–491.
51. Sayed, M.G., Ahmed, A.F., Ringold, J.R., Anderson, M.E., Bair, J.L., Mitros, F.A., Lynch, H.T., Tinley, S.T., Petersen, G.M., Giardiello, F.M., Vogelstein, B., and Howe, J.R. (2002) Germline SMAD4 or BMPR1A mutations and phenotype of juvenile polyposis. *Ann. Surg. Oncol.* **9**, 901–906.

52. Friedl, W., Uhlhaas, S., Schulmann, K., Stolte, M., Loff, S., Back, W., Mangold, E., Stern, M., Knaebel, H.P., Sutter, C., Weber, R.G., Pistorius, S., Burger, B., and Propping, P. (2002) Juvenile polyposis: massive gastric polyposis is more common in MADH4 mutation carriers than in BMPR1A mutation carriers. *Hum. Genet*. **111**, 108–111.
53. Gallione, C.J., Repetto, G.M., Legius, E., Rustgi, A.K., Schelley, S.L., Tejpar, S., Mitchell, G., Drouin, E., Westermann, C.J.J., and Marchuk, D.A. (2004) A combined syndrome of juvenile polyposis and hereditary haemorrhagic telanglectasia associated with mutations in MADH4 (SMAD4). *Lancet* **363**, 852–859.
54. Gallione, C.J., Richards, J.A., Letteboer, T.G.W., Rushlow, D., Prigoda, N.L., Leedom, T.P., Ganguly, A., Castells, A., van Amstel, J.K.P., Westermann, C.J.J., Pyeritz, R.E., and Marchuk, D.A. (2006) SMAD4 mutations found in unselected HHT patients. *J. Med. Genet.* **43**, 793–797.
55. Gallione, C., Aylsworth, A.S., Beis, J., Berk, T., Bernhardt, B., Clarks, R.D., Clericuzio, C., Danesino, C., Drautz, J., Fahl, J., Fan, Z., Faughnan, M.E., Ganguly, A., Garvie, J., Henderson, K., Kini, U., Leedom, T., Ludman, M., Lux, A., Maisenbacher, M., Mazzucco, S., Olivieri, C., van Amstel, J.K.P., Prigoda-Lee, N., Pyeritz, R.E., Reardon, W., Vandezande, K., Waldman, J.D., White, R.I., Williams, C.A., and Marchuk, D.A. (2010) Overlapping spectra of SMAD4 mutations in juvenile polyposis (JP) and JP-HHT syndrome. *Am. J. Med. Genet. A* **152A**, 333–339.
56. Sweet, K., Willis, J., Zhou, X.P., Gallione, C., Sawada, T., Alhopuro, P., Khoo, S.K., Patocs, A., Martin, C., Bridgeman, S., Heinz, J., Pilarski, R., Lehtonen, R., Prior, T.W., Frebourg, T., Teh, B.T., Marchuk, D.A., Aaltonen, L.A., and Eng, C. (2005) Molecular classification of patients with unexplained hamartomatous and hyperplastic polyposis. *JAMA* **294**, 2465–2473.
57. Howe, J.R., Haidle, J.L., Lal, G., Bair, J., Song, C., Pechman, B., Chinnathambi, S., and Lynch, H.T. (2007) ENG mutations in MADH4/BMPR1A mutation negative patients with juvenile polyposis. *Clin. Genet*. **71**, 91–92.
58. Kassouf, W., Ismail, H.R.A., Aprikian, A.G., and Chevalier, S. (2004) Whole-mount prostate sections reveal differential endoglin expression in stromal, epithelial, and endothelial cells with the development of prostate cancer. *Prostate Cancer Prostatic Dis*. **7**, 105–110.
59. Liu, Y.Q., Jovanovic, B., Pins, M., Lee, C., and Bergan, R.C. (2002) Overexpression of endoglin in human prostate cancer suppresses cell detachment, migration and invasion. *Oncogene* **21**, 8272–8281.
60. Lakshman, M., Huang, X., Ananthanarayanan, V., Jovanovic, B., Liu, Y., Craft, C.S., Romero, D., Vary, C.P., and Bergan, R.C. (2010) Endoglin suppresses human prostate cancer metastasis. *Clin. Exp. Metastasis* [Epub ahead of print]
61. Jovanovic, B.D., Huang, S., Liu, Y., Naguib, K.N., and Bergan, R.C. (2001) A simple analysis of gene expression and variability in gene arrays based on repeated observations. *Am. J. Pharmacogenomics* **1,** 145–152.
62. Wong, V.C.L., Chan, P.L., Bernabeu, C., Law, S., Wang, L.D., Li, J.L., Tsao, S.W., Srivastava, G., and Lung, M.L. (2008) Identification of an invasion and tumor-suppressing gene, endoglin (ENG), silenced by both epigenetic inactivation and allelic loss in esophageal squamous cell carcinoma. *Int. J. Cancer* **123**, 2816–2823.
63. Fernandez-Ruiz, E., St Jacques, S., Bellon, T., Letarte, M., and Bernabeu, C. (1993) Assignment of the human endoglin gene (End) to 9Q34→qter *Cytogenet. Cell Genet*. **64**, 204–207.
64. Yang, L.C., Leung, A.C.C., Ko, J.M.Y., Lo, P.H.Y., Tang, J.C.O., Srivastava, G., Oshimura, M., Stanbridge, E.J., Daigo, Y., Nakamura, Y., Tang, C.M.C., Lau, K.W., Law, S., and Lung, M.L. (2005) Tumor suppressive role of a 2.4Mb 9q33-q34 critical region and DEC1 in esophageal squamous cell carcinoma. *Oncogene* **24**, 697–705.
65. Leung, A.C.C., Wong, V.C.L., Yang, L.C., Chan, P.L., Daigo, Y., Nakamura, Y., Qi, R.Z., Miller, L.D., Liu, E.T.B., Wang, L.D., Li, J.L., Law, S., Tsao, S.W., and Lung, M.L. (2008) Frequent decreased expression of candidate tumor suppressor gene, DEC1, and its anchorage-independent growth properties and impact on global gene expression in esophageal carcinoma. *Int. J. Cancer* **122**, 587–594.
66. Henry, L., Johnson, D., Sarrió, D., Lee, S., Quinlan, P., Crook, T., Thompson, A., Reis-Filho, J.S, and Isacke, C.M. (2010) Endoglin expression in breast tumor cells suppresses invasion and metastasis and correlates with improved clinical outcome. *Oncogene* [Epub ahead of print]
67. Oxmann, D., Held-Feindt, J., Stark, A.M., Hattermann, K., Yoneda, T., and Mentlein, R. (2008) Endoglin expression in metastatic breast cancer cells enhances their invasive phenotype. *Oncogene* **27**, 3567–3575.
68. Pardali, E., van der Schaft, D.W.J., Wiercinska, E., Gorter, A., Hogendoorn, P.C.W., Griffioen, A.W., and ten Dijke, P. (2010) Critical role of endoglin in tumor cell plasticity of Ewing sarcoma and melanoma. *Oncogene* [Epub ahead of print]
69. Postiglione, L., Di Domenico, G., Caraglia, M., Marra, M., Giuberti, G., Del Vecchio, L., Montagnani, S., Macri, M., Bruno, E.M., Abbruzzese, A., and Rossi, G. (2005) Differential expression and cytoplasm/membrane distribution of endoglin (CD105) in human tumour cell lines: implications in the modulation of cell proliferation. *Int. J. Oncol*. **26**, 1193–1201.
70. Guerrero-Esteo, M., Lastres, P., Letamendia, A., Perez-Alvarez, M.J., Langa, C., Lopez, L.A., Fabra, A., Garcia-Pardo, A., Vera, S., Letarte, M., and Bernabeu, C. (1999) Endoglin overexpression modulates cellular morphology, migration, and adhesion of mouse fibroblasts. *Eur. J. Cell Biol*. **78**, 614–623.
71. Sanz-Rodriguez, F., Guerrero-Esteo, M., Botella, L.M., Banville, D., Vary, C.P.H., and Bernabeu, C. (2004) Endoglin regulates cytoskeletal organization through binding to ZRP-1, a member of the Lim family of proteins. *J. Biol. Chem*. **279**, 32858–32868.

72. Conley, B.A., Koleva, R., Smith, J.D., Kacer, D., Zhang, D.W., Bernabeu, C., and Vary, C.P.H. (2004) Endoglin controls cell migration and composition of focal adhesions -- function of the cytosolic domain. *J. Biol. Chem*. **279**, 27440–27449.
73. Lee, N.Y. and Blobe, G.C. (2007) The interaction of endoglin with beta-arrestin2 regulates transforming growth factor-beta-mediated ERK activation and migration in endothelial cells. *J. Biol. Chem*. **282**, 21507–21517.
74. Lee, N.Y., Ray, B., How, T., and Blobe, G.C. (2008) Endoglin promotes transforming growth factor beta-mediated Smad 1/5/8 signaling and inhibits endothelial cell migration through its association with GIPC. *J. Biol. Chem*. **283**, 32527–32533.
75. Ray, B.N., Lee, N.Y., How, T., and Blobe, G.C. (2010) ALK5 phosphorylation of the endoglin cytoplasmic domain regulates Smad1/5/8 signaling and endothelial cell migration. *Carcinogenesis* **31**, 435–441.
76. Craft, C.S., Romero, D., Vary, C.P.H., and Bergan, R.C. (2007) Endoglin inhibits prostate cancer motility via activation of the ALK2-Smad1 pathway. *Oncogene* **26**, 7240–7250.
77. Craft, C.S., Xu, L., Romero, D., Vary, C.P.H., and Bergan, R.C. (2008) Genistein induces phenotypic reversion of endoglin deficiency in human prostate cancer cells. *Mol. Pharmacol.* **73**, 235–242.
78. Romero, D., Terzic, A., Conley, B.A., Craft, C.S., Jovanovic, B., Bergan, R.C., and Vary, C.P.H. (2010) Endoglin phosphorylation by ALK2 contributes to the regulation of prostate cancer cell migration. *Carcinogenesis* **31**, 359–366.
79. Meng, Q.J., Lux, A., Holloschi, A., Li, J., Hughes, J.M.X., Foerg, T., McCarthy, J.E.G., Heagerty, A.M., Kioschis, P., Hafner, M., and Garland, J.M. (2006) Identification of Tctex2 beta, a novel dynein light chain family member that interacts with different transforming growth factor-beta receptors. *J. Biol. Chem*. **281**, 37069–37080.
80. Muenzner, P., Bachmann, V., Zimmermann, W., Hentschel, J., and Hauck, C.R. (2010) Human-restricted bacterial pathogens block shedding of epithelial cells by stimulating integrin activation. *Science* **239**, 1197–1201.
81. Wipff, J., Avouac, J., Borderie, D., Zerkak, D., Lemarechal, H., Kahan, A., Boileau, C., and Allanore, Y. (2008) Disturbed angiogenesis in systemic sclerosis: high levels of soluble endoglin. *Rheumatology* **47**, 972–975.
82. Venkatesha, S., Toporsian, M., Lam, C., Hanai, J., Mammoto, T., Kim, Y.M., Bdolah, Y., Lim, K.H., Yuan, H.T., Libermann, T.A., Stillman, I.E., Roberts, D., D'Amore, P.A., Epstein, F.H., Sellke, F.W., Romero, R., Sukhatme, V.P., Letarte, M., and Karumanchi, S.A. (2006) Soluble endoglin contributes to the pathogenesis of preeclampsia. *Nat. Med.* **12**, 642–649.
83. Li, C.G., Guo, B.Q., Wilson, P.B., Stewart, A., Byrne, G., Bundred, N., and Kumar, S. (2000) Plasma levels of soluble CD105 correlate with metastasis in patients with breast cancer. *Int. J. Cancer* **89**, 122–126.
84. Takahashi, N., Kawanishi-Tabata, R., Haba, A., Tabata, M., Haruta, Y., Tsai, H., and Seon, B.K. (2001) Association of serum endoglin with metastasis in patients with colorectal, breast, and ether solid tumors, and suppressive effect of chemotherapy on the serum endoglin. *Clin. Cancer Res*. **7**, 524–532.
85. Vo, M.N., Evans, M., Leitzel, K., Ali, S.M., Wilson, M., Demers, L., Evans, D.B., and Lipton, A. (2010) Elevated plasma endoglin (CD105) predicts decreased response and survival in a metastatic breast cancer trial of hormone therapy. *Breast Cancer Res. Treat*. **119**, 767–771.
86. Fujita, K., Ewing, C.M., Chan, D.Y.S., Mangold, L.A., Partin, A.W., Isaacs, W.B., and Pavlovich, C.P. (2009) Endoglin (CD105) as a urinary and serum marker of prostate cancer. *Int. J. Cancer* **124**, 664–669.
87. Karam, J.A., Svatek, R.S., Karakiewicz, P.I., Gallina, A., Roehrborn, C.G., Slawin, K.M., and Shariat, S.F. (2008) Use of preoperative plasma endoglin for prediction of lymph node metastasis ion patients with clinically localized prostate cancer. *Clin. Cancer Res*. **14**, 1418–1422.
88. Svatek, R.S., Karam, J.A., Roehrborn, C.G., Karakiewicz, P.I., Slawin, K.M., and Shariat, S.F. (2008) Preoperative plasma endoglin levels predict biochemical progression after radical prostatectomy. *Clin. Cancer Res*. **14**, 3362–3366.
89. Calabro, L., Fonsatti, E., Bellomo, G., Alonci, A., Colizzi, F., Sigalotti, L., Altomonte, M., Musolino, C., and Maio, M. (2003) Differential levels of soluble endoglin (CD105) in myeloid malignancies. *J. Cell. Physiol*. **194**, 171–175.
90. Mysliwiec, P., Pawlak, K., Bandurski, R., and Kedra, B. (2009) Soluble angiogenesis markers in gastric tumor patients. *Folia Histochem. Cytobiol*. **47**, 81–86.
91. Mysliwiec, P., Pawlak, K., Kuklinski, A., and Kedra, B. (2008) Combined perioperative plasma endoglin and VEGF-A assessment in colorectal cancer patients. *Folia Histochem. Cytobiol.* **46**, 487–492.
92. Odegaard, E., Davidson, B., Engh, V., Onsrud, M., and Staff, A.C. (2008) Assessment of endoglin and calprotectin as potential biomarkers in ovarian carcinoma and borderline tumors of the ovary. *Am. J. Obstet. Gynecol*. **199**, 533.e1–533.e8.
93. Furstenberger, G., von Moos, R., Lucas, R., Thurlimann, B., Senn, H.J., Hamacher, J., and Boneberg, E.M. (2006) Circulating endothelial cells and angiogenic serum factors during neoadjuvant chemotherapy of primary breast cancer. *Br. J. Cancer* **94**, 524–531.
94. Bellone, G., Solerio, D., Chiusa, L., Brondino, G., Carbone, A., Prati, A., Scirelli, T., Camandona, M., Palestro, G., and Poli, M.D. (2007) Transforming growth factor-beta binding receptor endoglin (CD105) expression in esophageal cancer and in adjacent nontumorous esophagus as prognostic predictor of recurrence. *Ann. Surg. Oncol*. **14**, 3232–3242.

95. Hawinkels, L.J.A.C., Kuiper, P., Wiercinska, E., Verspaget, H.W., Liu, Z., Pardali, E., Sier, C.F.M., and ten Dijke, P. (2010) Matrix metalloproteinase-14 (MT1-MMP)-mediated endoglin shedding inhibits tumor angiogenesis. *Cancer Res.* **70**, 4141–4150.
96. Fonsatti, E., Altomonte, M., Nicotra, M.R., Natali, P.G., and Maio, M. (2003) Endoglin (CD105): a powerful therapeutic target on tumor-associated angiogenetic blood vessels. *Oncogene* **22**, 6557–6563.
97. Gingery, A., Bahe, E.L., and Gilbert, J.S. (2009) Placental ischemia and breast cancer risk after preeclampsia: tying the knot. *Expert Rev. Anticancer Ther*. **9**, 671–681.
98. Yuspa, S.H. (1998) The pathogenesis of squamous cell cancer: lessons learned from studies of skin carcinogenesis. *J. Dermatol. Sci*. **17**, 1–7.
99. Kemp, C.J. (2005) Multistep skin cancer in mice as a model to study the evolution of cancer cells. *Semin. Cancer Biol*. **15**, 460–473.
100. Abel, E.L., Angel, J.M., Kiguchi, K., and DiGiovanni, J. (2009) Multi-stage chemical carcinogenesis in mouse skin: fundamentals and applications. *Nat. Protoc*. **4**, 1350–1362.
101. Luch, A. (2005) Nature and nurture -- lessons from chemical carcinogenesis. *Nat. Rev. Cancer* **5**, 113–125.
102. Quintanilla, M., Brown, K., Ramsden, M., and Balmain, A. (1986) Carcinogen-specific mutation and amplification of Ha-Ras during mouse skin carcinogenesis. *Nature* **322**, 78–80.
103. Glick, A., Ryscavage, A., Perez-Lorenzo, R., Hennings, H., Yuspa, S., and Darwiche, N. (2007) The high-risk benign tumor: evidence from the two-stage skin cancer model and relevance for human cancer. *Mol. Carcinog*. **46**, 605–610.
104. Klein-Szanto, A.J. (1997) Neoplastic and preneoplastic lesions of the mouse skin. In *Pathology of Neoplasia and Preneoplasia in Rodents* **359**, 1–19.
105. Akhurst, R.J. and Balmain, A. (1999) Genetic events and the role of TGF-beta in epithelial tumour progression. *J. Pathol*. **187**, 82–90.
106. Li, A.G.Q., Lu, S.L., Han, G.W., Hoot, K.E., and Wang, X.J. (2006) Role of TGF beta in skin inflammation and carcinogenesis. *Mol. Carcinog*. **45**, 389–396.
107. Cui, W., Fowlis, D.J., Bryson, S., Duffie, E., Ireland, H., Balmain, A., and Akhurst, R.J. (1996) TGF-beta1 inhibits the formation of benign skin tumors, but enhances progression to invasive spindle carcinomas in transgenic mice. *Cell* **86**, 531–542.
108. Quintanilla, M., Ramirez, J.R., Perez-Gomez, E., Romero, D., Velasco, B., Letarte, M., Lopez-Novoa, J.M., and Bernabeu, C. (2003) Expression of the TGF-beta coreceptor endoglin in epidermal keratinocytes and its dual role in multistage mouse skin carcinogenesis. *Oncogene* **22**, 5976–5985.
109. Jerkic, M., Rodriguez-Barbero, A., Prieto, M., Toporsian, M., Pericacho, M., Rivas-Elena, J.V., Obreo, J., Wang, A., Perez-Barriocanal, F., Arevalo, M., Bernabeu, C., Letarte, M., and Lopez-Novoa, J.M. (2006) Reduced angiogenic responses in adult endoglin heterozygous mice. *Cardiovasc. Res*. **69**, 845–854.
110. Perez-Gomez, E., Villa-Morales, M., Santos, J., Fernandez-Piqueras, J., Gamallo, C., Dotor, J., Bernabeu, C., and Quintanilla, M. (2007) A role for endoglin as a suppressor of malignancy during mouse skin carcinogenesis. *Cancer Res*. **67**, 10268–10277.
111. Mercado-Pimentel, M.E., Hubbard, A.D., and Runyan, R.B. (2007) Endoglin and Alk5 regulate epithelial-mesenchymal transformation during cardiac valve formation. *Dev. Biol*. **304**, 420–432.
112. Haddow, S., Fowlis, D.J., Parkinson, K., Akhurst, R.J., and Balmain, A. (1991) Loss of growth-control by TGF-beta occurs at a late stage of mouse skin carcinogenesis and is independent of Ras gene activation. *Oncogene* **6**, 1465–1470.
113. Santibanez, J.F., Pérez-Gómez, E., Fernandez-L, A., Garrido-Martin, E.M., Carnero, A., Malumbres, M., Vary, C.P., Quintanilla, M., and Bernabéu, C. (2010) The TGF-beta co-receptor endoglin modulates the expression and transforming potential of H-Ras. *Carcinogenesis* [Epub ahead of print]
